# Disease surveillance in albatrosses and petrels from the Southwest Atlantic and Southern Ocean

**DOI:** 10.1017/S0031182025100590

**Published:** 2025-09

**Authors:** Patricia Pereira Serafini, Annelise Zabel Sgarioni, Richard A. Phillips, Alice Pereira, Tiffany Emmerich, Thamires P. Pontes, Derek B. Amorim, Cristiane K. M. Kolesnikovas, André O. S. Lima, Guilherme Klafke, José Reck, Afonso C. D. Bainy, Karim H. Lüchmann, Camille Bonneaud

**Affiliations:** 1 Laboratório de Biomarcadores de Contaminação Aquática e Imunoquímica, Universidade Federal de Santa Catarina – UFSC, Florianópolis, SC, Brazil; 2 Centro Nacional de Pesquisa e Conservação de Aves Silvestres, Instituto Chico Mendes de Conservação da Biodiversidade – ICMBio, Florianópolis, SC, Brazil; 3 Laboratório de Genética Molecular, Escola Politécnica, Universidade do Vale do Itajaí (UNIVALI), Itajaí, SC, Brazil; 4 British Antarctic Survey, Natural Environment Research Council, Cambridge, Cambridgeshire, UK; 5 Projeto Albatroz, Florianópolis, SC, Brazil; 6 Unidade de Estabilização de Animais Marinhos, Universidade do Vale de Itajaí (UNIVALI), Penha, SC, Brazil; 7 Centro de Estudos Costeiros, Limnológicos e Marinhos (CECLIMAR), Universidade Federal do Rio Grande do Sul (UFRGS), Imbé, RS, Brazil; 8 Associação R3 Animal, Florianópolis, SC, Brazil; 9 Laboratório de Parasitologia, Instituto de Pesquisas Veterinárias Desidério Finamor (IPVDF), Eldorado do Sul, RS, Brazil; 10 Departamento de Educação Científica e Tecnológica, Universidade do Estado de Santa Catarina – UDESC, Florianópolis, SC, Brazil; 11 Centre for Ecology and Conservation, University of Exeter, Penryn, UK

**Keywords:** climate-driven risks, disease surveillance, haemosporidian parasites, seabirds health, vector-borne pathogens

## Abstract

Emerging infectious diseases pose threats to wildlife, particularly in geographically isolated populations where hosts may lack prior exposure and immunity. Seabirds inhabiting remote islands in the southwest Atlantic and Southern Ocean, including threatened albatrosses and petrels, are increasingly affected by infectious pathogens. However, baseline data on vector-borne infections in these species remain scarce. This study assessed the presence of vector-borne haemosporidian parasites (*Plasmodium, Haemoproteus* and *Leucocytozoon*) and bacterial pathogens (*Borrelia burgdorferi sensu lato, Anaplasma* and *Ehrlichia*) in albatrosses and petrels, providing insights into disease prevalence and potential threats to these populations. We analysed blood and tissue samples from 269 individuals of 5 albatross and 12 petrel species, collected over an 11-year period (2013–2023) from South Georgia and multiple sites along the Brazilian coastline. Molecular assays, including nested Polymerase Chain Reaction (PCR), were used for pathogen screening. Blood smears from birds sampled in South Georgia were also examined for haemoparasites via light microscopy. We found no molecular or microscopy evidence of infection with haemosporidian parasites, *Borrelia, Anaplasma* or *Ehrlichia* in any of the samples. These findings suggest that vector-borne pathogens are either absent or at low prevalence, possibly because of limited vector presence, natural resistance or historical isolation from infection. Continuous monitoring is critical given current environmental changes and risks of pathogen introduction via climate-driven shifts in vector distribution. Our study establishes an essential baseline for future disease surveillance, prevention and mitigation in albatrosses and petrels, underscoring the importance of long-term monitoring to detect emerging pathogens in vulnerable seabird populations.

## Introduction

Emerging infectious diseases can have devastating impacts on hosts, sometimes with consequences for entire ecosystems (Machalaba et al., 2020; Nicholson et al., 2020; Ferreyra et al., 2022; Jain, 2023). For example, the pandemic of high pathogenicity avian influenza (HPAI H5Nx) resulted in the death of over half a billion wild birds and poultry worldwide within just a few years of emergence in the early 2020s (Gamarra-Toledo et al., 2023; Roberts et al., 2023; Puryear and Runstadler, 2024). Diseases can be particularly severe in areas where hosts are shielded geographically from pathogen exposure, particularly those on remote islands (Wyatt et al., 2008; Atkinson and LaPointe, 2009; Vanstreels et al., 2023). Such threats to isolated populations are often exacerbated by anthropogenic effects, such as global connectivity and climate change, in part through effects on pathogen and vector survival, distribution and spread (Steig et al., 2009; Lynton-Jenkins et al., 2021). Monitoring the health and infection status of vulnerable species inhabiting remote locations can therefore be critical for their conservation.

The islands of the southwest Atlantic and Southern Ocean are home to a rich diversity of bird species, including many endemics or globally important breeding populations (Poncet et al., 2020; Favero et al., 2024; Poncet et al., 2017). This includes highly threatened albatrosses and petrels, many of which are declining because of incidental mortality (bycatch) in fisheries, predation by invasive species, climate change, or degradation/loss of nesting habitat (Dias et al., 2019; Phillips et al., 2023; Baker et al., 2024). Until recently, there have been few documented cases of mass mortality on islands where albatrosses and petrels breed, but it is unclear whether this is because these populations are largely shielded from infection (Vanstreels et al., 2023), or because of a lack of attention to their health and disease status (Uhart et al., 2018). Avian cholera (*Pasteurella multocida*) is, however, thought to have caused mortality in albatrosses and petrels in the 1980s, with more recent infections in asymptomatic individuals raising questions around the reservoir potential of these species (Gamble et al., 2019). Overall, outbreaks of infectious diseases in albatrosses and petrels appear to be on the rise, e.g. avian cholera and *Erysipelothrix rhusiopathiae* cause yearly recurrent die-offs of the Indian yellow-nosed albatross (*Thalassarche carteri*) on Amsterdam Island (Jaeger et al., 2020). In 2024, an outbreak of HPAI H5N1 on South Georgia caused mortality in multiple taxa, including wandering albatrosses (*Diomedea exulans*), which are listed as Vulnerable by the International Union for the Conservation of Nature, as well as brown skuas (*Stercorarius antarcticus*), gentoo penguins (*Pygoscelis papua*) and Antarctic fur seals (*Arctocephalus gazella*) (Bennison et al., 2024). Given increasing likelihood of disease outbreaks at breeding sites (Banyard et al., 2024), the establishment of continuous monitoring programmes of albatross and petrel health is integral to the rapid detection of emerging diseases and effective predictions of disease spread based on prior infection history (Vanstreels et al., 2023).

Infectious pathogens at risk of emergence in seabirds breeding on remote islands include vector-borne haemosporidian parasites in the genera *Plasmodium, Haemoproteus* and *Leucocytozoon* (Quillfeldt et al., 2011; Parsons et al., 2017; Vanstreels et al., 2018; Muñoz-Leal et al., 2019), which cause avian malaria or malaria-like diseases (Valkiunas, 2005; Palinauskas et al., 2008; Bensch et al., 2009; Pacheco and Escalante, 2023). Infections with these parasites have been associated with increased mortality, population declines and even extinctions (Warner, 1968; Hill et al., 2010). Sublethal effects include reductions in body condition, sexual ornamentation or reproductive success, as well as prolonged stopovers or delayed migration (Martínez-Abraín et al., 2004; Marzal et al., 2005, 2008; Barbosa and Palacios, 2009; Quillfeldt et al., 2011; Hegemann et al., 2018). In seabirds, infection with *Plasmodium relictum, P. circumflexum* and *P. vaughani* has been associated with increased mortality in captive Humboldt and Magellanic penguins (*Spheniscus demersus* and *S. magellanicus*), little penguins (*Eudyptula minor*) and Atlantic puffins (*Fratercula arctica*) (Sallaberry-Pincheira et al., 2015; Sijbranda et al., 2017; Meister et al., 2021).

Other haemoparasites isolated recently from seabirds at risk of emergence include the bacteria *Borrelia* (Dietrich et al., 2011; Schramm et al., 2014; Parsons et al., 2017; Vanstreels et al., 2023), *Anaplasma* and *Ehrlichia* (Anaplasmataceae) (Vanstreels et al., 2018; Muñoz-Leal et al., 2019). These parasites are transmitted through ticks such as *Ixodes* spp. and *Argasidae* spp., which are common in many seabird colonies (Dietrich et al., 2011, 2014; Vanstreels et al., 2018; Muñoz-Leal et al., 2019). *Borrelia* parasites have been isolated from *Ixodes uriae* ticks collected in colonies of razorbills (*Alca torda*), Atlantic puffins (*F. arctica*) and black-browed albatrosses (*Thalassarche melanophris*) (Olsen et al., 1993, 1995; Gylfe et al., 1999; Munro et al., 2019). Such infections can be pathogenic in seabirds (Yabsley et al., 2012; Parsons et al., 2018; Vanstreels et al., 2019), as evidenced by a *Borrelia*-positive African penguin (*S. demersus*) that presented antemortem neurological signs and lesions similar to those reported in an owl fatally infected with *B. hermsii* and a domestic fowl infected with *B. anserina* (Dickie and Barrera, 1964; Thomas et al., 2002; Bunikis et al., 2004; Yabsley et al., 2012). *Anaplasma phagocytophilum* has been detected in blood samples from passerines, suggesting their potential role in transmitting the bacterium to ticks; however, the significance of wild birds in the infectious cycle of this parasite remains unclear (Pedersen et al., 2020). Although only mammals have so far been confirmed as competent hosts and reservoirs for *Ehrlichia* bacteria (Rar and Golovljova, 2011), there is growing evidence that the host range may extend to other vertebrates after the detection in wild birds in Brazil of *Ehrlichiae* typically associated with ungulates and carnivores (Machado et al., 2012).

The goal of this study was to survey vector-borne infectious diseases in albatrosses and petrels sampled in the southwest Atlantic and Southern Ocean, with the aim of detecting any novel infection, as well as establishing baselines for future surveillance. We screened 5 species of albatrosses and 12 species of petrels for the presence of haemosporidian parasites (*Plasmodium, Haemoproteus* and *Leucocytozoon*), and the bacterial pathogens *Anaplasma, Ehrlichia* and *B. burgdorferi sensu lato* (s.l.). Understanding host–pathogen interactions in these species will be key to informing conservation strategies of these threatened species and mitigating potential disease threats in a rapidly changing environment.

## Materials and methods

### Sampling

The samples were obtained between 2013 and 2023 from 4 different sources: (i) breeding adults and chicks at colonies on Bird Island, South Georgia (54°00′S, 38°03′W); (ii) seabirds bycaught in Brazilian fisheries; (iii) birds found in a weakened state on the Brazilian coast and taken to 2 rehabilitation centres; and (iv) seabirds monitored in stranding networks in Brazil (Table 1). Detailed fieldwork procedures, methods of sampling and locations of seabirds surveyed are provided in Supplementary Table S1.

**Table 1.** Albatrosses and petrels screened for vector-borne parasites from 2013 to 2023 from the southern Brazilian Coast, Brazilian fisheries and from colonies in Bird Island, South Georgia

Family	Species	Sampling location	No. of individuals tested	Haemoparasites investigated
Diomedeidae	Wandering albatross *Diomedea exulans*	Bird Island, South Georgia	50	*Plasmodium, Haemoproteus, Leucocytozoon, Borrelia burgdorferi s.l.*
	Tristan albatross	Rehabilitation centres and fisheries bycaught birds, Brazil	1	*Anaplasma, Ehrlichia*
	*Diomedea dabbenena*			
	Southern royal albatross	Rehabilitation centres and fisheries bycaught birds, Brazil	1	*Anaplasma, Ehrlichia*
	*Diomedea epomophora*			
	Atlantic yellow-nosed albatross	Rehabilitation centres and fisheries bycaught birds, Brazil	15	*Anaplasma, Ehrlichia*
	*Thalassarche chlororhynchos*			
	Black-browed albatross	Rehabilitation centres, stranding network and fisheries bycaught birds, Brazil	29	*Plasmodium, Haemoproteus, Anaplasma, Ehrlichia*
	*Thalassarche melanophris*			
Procellariidae	Great shearwater	Rehabilitation centres and fisheries bycaught birds, Brazil	6	*Anaplasma, Ehrlichia*
	*Ardenna gravis*			
	Sooty shearwater	Rehabilitation centres and fisheries bycaught birds, Brazil	2	*Anaplasma, Ehrlichia*
	*Ardenna grisea*			
	Cory’s shearwater	Rehabilitation centres and fisheries bycaught birds, Brazil	10	*Anaplasma, Ehrlichia*
	*Calonectris borealis*			
	Cape petrel	Rehabilitation centres and fisheries bycaught birds, Brazil	3	*Anaplasma, Ehrlichia*
	*Daption capense*			
	Southern fulmar	Rehabilitation centres and fisheries bycaught birds, Brazil	1	*Anaplasma, Ehrlichia*
	*Fulmarus glacialoides*			
	Southern giant petrel	Rehabilitation centres and fisheries bycaught birds, Brazil	8	*Anaplasma, Ehrlichia*
	*Macronectes giganteus*			
	Northern giant petrel	Rehabilitation centres and fisheries bycaught birds, Brazil; Bird Island, South Georgia	33	*Anaplasma, Ehrlichia*
				*Plasmodium, Haemoproteus, Leucocytozoon, Borrelia burgdorferi s.l.*
	White-chinned petrel	Rehabilitation centres and fisheries bycaught birds, Brazil; Bird Island, South Georgia	61	*Anaplasma, Ehrlichia*
	*Procellaria aequinoctialis*			*Plasmodium, Haemoproteus, Leucocytozoon, Borrelia burgdorferi s.l.*
	Atlantic petrel	Rehabilitation centres and fisheries bycaught birds, Brazil	1	*Anaplasma, Ehrlichia*
	*Pterodroma incerta*			
	Soft-plumaged petrel	Rehabilitation centres and fisheries bycaught birds, Brazil	1	*Anaplasma, Ehrlichia*
	*Pterodroma mollis*			
	Manx shearwater	Rehabilitation centres and fisheries bycaught birds, Brazil	42	*Anaplasma, Ehrlichia*
	*Puffinus puffinus*			
Oceanitidae	Wilson’s storm petrel	Rehabilitation centres and fisheries bycaught birds, Brazil	5	*Anaplasma, Ehrlichia*
	*Oceanites oceanicus*			

### DNA extraction and molecular analysis

A total of 96 DNA samples from the seabirds sampled in South Georgia were extracted using a QIAamp DNA Micro Kit (Qiagen, Hilden, Germany) following the manufacturer’s protocol. For all other samples (Table 1), DNA was extracted using the phenol-chloroform method (Sambrook et al., 1989), with the exception of blood samples preserved in ethanol (Supplementary Table S1), which were extracted using a slightly modified approach (Maia et al., 2017). First, we tested for the presence of haemosporidians (*Plasmodium, Haemoproteus* and *Leucocytozoon*) in a subset of samples (*N* = 96 blood samples from South Georgia and 30 liver samples), using a nested PCR targeting a 617-bp fragment of the cytochrome b gene (Hellgreen et al., 2004) and including DNA from *Plasmodium gallinaceum* strain A8 (MalAvi lineage GALLUS01), *Haemoproteus majoris* (MalAvi lineage PARUS1) and *Leucocytozoon* (MalAvi lineage PARUS22) as positive controls. Second, we tested for the presence of *Borrelia burgdorferi sensu lato* (s.l.) using a nested PCR, which targets the 5S–23S rRNA spacer region of all *Borreliae* species and the same positive controls mentioned by Newman et al. (2015). Finally, we tested for the presence of *Ehrlichia* and *Anaplasma* in 219 tissue samples using a PCR protocol specifically designed to amplify a 345-bp fragment of the 16S rRNA gene (Parola et al., 2000); *Anaplasma marginale* DNA was used as a positive control. In all assays, we included ultrapure water as negative controls.

### Light microscopy of blood smears

The 96 blood smears taken from the live seabirds at South Georgia were analysed by light microscopy, following Merino et al. (1997) and Quillfeldt et al. (2010). In brief, one half of the slide was scanned at ×200 magnification to look for haemoparasites, and at least 20 fields in the other half at ×400 to look for intracellular stages of haematozoa. A minimum of 2000 to 10 000 erythrocytes were also checked using the oil immersion objective (1000×).

## Results

None of the parasites of interest were detected in the 269 sampled seabirds (Table 1). This included no evidence of infection with either *Plasmodium, Haemoproteus* or *Leucocytozoon* parasites in the 96 samples from live albatrosses and petrels obtained in South Georgia, nor of infection with *Plasmodium* or *Haemoproteus* in the liver samples from wandering albatross chicks (18) or black-browed albatrosses (12) found dead at South Georgia or in southern Brazil. We found no evidence of infection with *Borrelia* spp. (Table 1) in any of the 96 samples from live albatrosses and petrels obtained in South Georgia, or with *Anaplasmataceae* parasites in any of the 219 tissue samples from seabirds found at the Brazilian coast or obtained as bycatch. Microscopic observations were consistent with molecular findings in that we did not detect the presence of parasitized erythrocytes in any of the 96 blood smears obtained from live wandering albatrosses, northern giant petrels and white-chinned petrels at South Georgia.

## Discussion

Molecular and microscopy methods were used to screen for the presence of key blood parasites in a total of 269 birds from 5 species of albatrosses and 12 species of petrels sampled in the southwest Atlantic and Southern Ocean over a period of 11 years (2013–2023). None of the samples were found to be infected with parasites of the genera *Plasmodium, Leucocytozoon, Haemoproteus, Borrelia, Anaplasma* or *Ehrlichia*. Although detecting parasites in blood smears through microscopy is challenging when infection intensity is low (Valkiunas, 2005), the high sensitivity of molecular methods increases our confidence that none of the birds were infected. These results are consistent with other investigations of blood parasite infections conducted in seabirds of the Antarctic region (Laird, 1961; Quillfeldt et al., 2010; Llanos et al., 2018). Screening of 455 birds from 14 species sampled between 1975 and 1978 in South Georgia found that none of the samples tested positive for blood parasites, except a small proportion of wandering, black-browed and grey-headed albatrosses, which were infected with the previously undescribed *Hepatozoan albatrossi* (Peirce and Prince, 1980). Documented cases of *Hepatozoon* in the Antarctic and subantarctic regions are limited to *Hepatozoon albatrossi*, reported in albatrosses and storm petrels (Merino et al., 2014; Parsons et al., 2017). Our study aimed to screen for other parasites with poorly known occurrence, and excluded *Hepatozoon* because its presence is already established. Our findings provide further evidence that most seabird species are likely relatively free of blood parasites (Quillfeldt et al., 2011).

The lack of blood parasites in the samples may be explained partly by the harsh environmental conditions at the breeding grounds, which may preclude vector persistence (Martínez-Abraín et al., 2004). To date, there are no records of vectors such as hematophagous ceratopogonid or hippoboscid biting flies, or *Aedes* mosquitoes, in subantarctic or Antarctic islands where most of the study species nest (Quillfeldt et al., 2011; Ferreira et al., 2020). Such vectors may, however, occur in feeding areas, making disease transmission possible from infected coastal seabird or land bird species. Infection at non-breeding grounds is thought to drive the incidence of *Leucocytozoon* and *Plasmodium* lineages in Caspian gulls (*Larus cachinnans*) wintering on the coast of Poland (Zagalska-Neubauer and Bensch, 2016), and of *Haemoproteus* spp. in Manx shearwaters and black-browed albatrosses off the coast of Brazil (Sgarioni et al., 2024), since these species tested negative at their breeding colonies (Quillfeldt et al., 2011). The petrel and albatross species that were sampled in South Georgia, including white-chinned petrels, wandering albatrosses and northern giant petrels, can travel to the Patagonian Shelf or shelf-break to feed during the breeding season, and stay there for part or all of the nonbreeding season (Phillips et al., 2006; González‐Solís et al., 2007; Froy et al., 2015; Clay et al., 2019; Granroth-Wilding and Phillips, 2019). The lower latitudes of the Patagonian Shelf and shelf-break, compared to the Antarctic and sub-Antarctic regions, increase the likelihood of vector presence and infection risk, with the potential for subsequent transmission by returning migrants to colonies during the breeding season (Quillfeldt et al., 2011; Sallaberry-Pincheira et al., 2015). Indeed, haemosporidian parasites, such as *P. relictum, P. circumflexum* and *P. vaughani*, as well as *Borrelia* parasites, can be found in migratory birds (McDiarmid, 1969; Wolcott et al., 2021; Bennett et al., 2024), and seabirds are thought to play a role in the dispersal of *Anaplasma* and *Ehrlichia* bacteria (Vanstreels et al., 2018; Muñoz-Leal et al., 2019). As such, the routine surveillance for blood parasite infections in seabirds in the southwest Atlantic and Southern Ocean is essential for detecting any shifts of disease vectors to higher latitudes, thereby increasing risks of range expansions for vector-borne parasites.

While the lack of detectable blood parasites is reassuring, seabirds of the southwest Atlantic and Southern Ocean remain at risk of future infections. *Ehrlichia* spp. was recorded recently in southern Chile in an *I. uriae* tick (Muñoz-Leal et al., 2019), which is a common parasite of seabirds on Antarctic and subantarctic islands (Vanstreels et al., 2020), and new *Plasmodium* infections were recently recorded in seabirds in New Zealand, including in a procellariiform species, the Westland petrel (*Procellaria westlandica*) (Bennett et al., 2024). Many albatrosses and petrels already face numerous other threats, so novel infections are potentially serious additional risks (Heard et al., 2013; Dias et al., 2019; Phillips et al., 2023). As a result, developing conservation and management plans for seabirds of the southwest Atlantic and Southern Ocean will require the implementation of proactive risk assessments, biosecurity measures and long-term disease surveillance, particularly at important breeding and non-breeding areas (Uhart et al., 2018). We recommend that surveillance programmes of blood parasite infections go beyond relying only on visual observations of diseased birds, and incorporate active screenings of seabird samples.

## Supporting information

Pereira Serafini et al. supplementary material Pereira Serafini et al. supplementary material

## Supplementary material

The supplementary material for this article can be found at https://doi.org/10.1017/S0031182025100590.
