# Supplementary material for: Disease surveillance in albatrosses and petrels from the Southwest Atlantic and Southern Ocean
Source: Parasitology. 2025 Aug 11;152(11):1172–8. doi: 10.1017/S0031182025100590 (PMC12921255; doi:10.1017/S0031182025100590)
Supplement: Pereira Serafini et al. supplementary material [file S0031182025100590sup001.docx]

**Supplementary Material**

Disease surveillance in albatrosses and petrels from the southwest Atlantic and Southern Ocean

Patricia P. Serafini^1,2^, Annelise Z. Sgarioni^3^, Richard A. Phillips^4^, Alice Pereira^5^, Tiffany Emmerich^6^, Thamires P. Pontes^6^, Derek B. Amorim^7^, Cristiane M. Kolesnikovas^8^, André O. S. Lima^3^, Guilherme Klafke^9^, José Reck^9^, Afonso C.D. Bainy^1^, Karim H. Lüchmann^10^, and Camille Bonneaud^11^

**Materials and methods**

Detailed Sampling and Fieldwork

The samples were obtained between 2013 and 2023 from four different sources. First, fieldwork was carried out on Bird Island, South Georgia (54°00′ S, 38°03′ W), where seabirds were captured and sampled under permit during the austral summer 2014/15 (Figure 1). Blood samples were obtained from 32 adults of each of the following three species: wandering albatross (*Diomedea exulans*), northern giant petrel (*Macronectes halli*) and white-chinned petrel (*Procellaria aequinoctialis*). A few drops of blood were drawn for each individual using a sterile needle and stored on an FTA card (Whatman FTA card technology, Sigma Aldrich, Darmstadt, Germany). In addition, blood smears were made in the field on microscope slides, which were later fixed with 100% methanol and stained with Giemsa (Merck, Darmstadt, Germany). Second, samples of blood (N=85), liver (N=92) and spleen (N=42) were collected from 143 individuals belonging to 16 procellariform species obtained either from (i) a biobank “Banco Nacional de Amostras Biologicas de Albatrozes e Petreis” (BAAP), which holds samples of birds bycaught in Brazilian fisheries and from seabirds stranding networks in Brazil, or (ii) birds found in a weakened state on the Brazilian coast and taken to two rehabilitation centers: “Unidade de Estabilização de Animais Marinhos” from Universidade do Vale de Itajai (UNIVALI) in Penha municipality, Santa Catarina state (48°36′ W; 26°46′ S), and the “Centro de Estudos Costeiros, Limnológicos e Marinhos” (CECLIMAR) from Universidade Federal do Rio Grande do Sul in Imbe municipality, Rio Grande do Sul state (50°8′ W; 29°58′ S). Third, we obtained liver samples in RNALater (Thermo Fisher Scientific, Waltham, MA, USA) from 18 wandering albatross chicks that died of natural causes on Bird Island between 2020 and 2021. Fourth, we obtained twelve liver samples in RNALater from black-browed albatrosses (*Thalassarche melanophris*) found dead on the southern coast of Brazil by members of “Projeto de Monitoramento de Praias da Bacia de Santos” (PMP-BS) between 2017 and 2023 (Figure 1). After collection, all tissues samples were maintained frozen at – 20 °C until analysis or stored at – 80 °C until further investigation.

In total, we analysed samples from: (i) 96 seabirds from the family Diomedeidae, including wandering albatross (N= 50), Tristan albatross (*Diomedea dabbenena*, N=1), southern royal albatross (*Diomedea epomophora*, N=1), Atlantic yellow-nosed albatross (*Thalassarche chlororhynchos*, N=15), black-browed albatross (N=29); 173 seabirds from the family Procellariidae, including great shearwater (*Ardenna gravis*, N=6), sooty shearwater (*Ardenna grisea*, N=2), Cory's shearwater (*Calonectris borealis*, N=10), Cape petrel (*Daption capense*, N=3), southern fulmar (*Fulmarus glacialoides*, N=1), southern giant petrel (*Macronectes giganteus*, N=8), northern giant petrel (N=33), white-chinned petrel (N=61), Atlantic petrel (*Pterodroma incerta*, N=1), soft-plumaged petrel (*Pterodroma mollis*, N=1), Manx shearwater (*Puffinus puffinus*, N=42); and 5 seabirds from the family Oceanitidae, all of which were Wilson's storm petrels (*Oceanites oceanicus*).

| Table S1. List of samples from albatrosses and petrels obtained from the southern Brazilian coast, Brazilian fisheries and South Georgia from 2013 to 2023 that were screened for vector-borne parasites. | | | | | | | | | | | | |
| --- | --- | --- | --- | --- | --- | --- | --- | --- | --- | --- | --- | --- |
| **Family (number of individuals tested)** | | | | **Species (number of individuals tested** | | **Sampling location and dates** | **Type of sample** | **Nº of samples analyzed** | | **Hemoparasites investigated** | **Detection method** | **Test laboratory** |
| **Diomedeidae**  **(n = 96)** | | | | Wandering albatross  *Diomedea exulans*  (n = 50) | | Bird Island, South Georgia  2014/2015 season | Blood (in FTA cards) and blood smears (on microscope slides) | 32 | *Plasmodium, Haemoproteus, Leucocytozoon, Borrelia burgdorferi s.l.* | | Molecular analysis and Blood smear | Molecular biology lab of the University of Exeter |
|  | | |  | | Bird Island, South Georgia  2020/2021 season | | Liver (in RNA later) | 18 | *Plasmodium, Haemoproteus* | | Molecular analysis | LABCAI of the Federal University of Santa Catarina state and parasitology lab of the IPVDF |
|  | | Tristan albatross  *Diomedea dabbenena*  (n = 1) | | | Rehabilitation centers, Brazil (UNIVALI, CECLIMAR), and BAAP | | Blood (frozen and in ethanol) and liver (frozen) | 2 | *Anaplasma, Ehrlichia* | | Molecular analysis | Parasitology lab of the IPVDF |
|  | | Southern royal albatross  *Diomedea epomophora*  (n = 1) | | | Rehabilitation centers, Brazil (UNIVALI, CECLIMAR), and BAAP | | Blood (frozen and in ethanol) and liver (frozen) | 2 | *Anaplasma, Ehrlichia* | | Molecular analysis | Parasitology lab of the IPVDF |
|  | | Atlantic yellow-nosed albatross  *Thalassarche chlororhynchos*  (n = 15) | | | Rehabilitation centers, Brazil (UNIVALI, CECLIMAR), and BAAP | | Blood (frozen and in ethanol) | 13 | *Anaplasma, Ehrlichia* | | Molecular analysis | Parasitology lab of the IPVDF |
|  | |  | | | Rehabilitation centers, Brazil (UNIVALI, CECLIMAR), and BAAP | | Liver (frozen) | 8 | *Anaplasma, Ehrlichia* | | Molecular analysis | Parasitology lab of the IPVDF |
|  | |  | | | Rehabilitation centers, Brazil (UNIVALI, CECLIMAR), and BAAP | | Spleen (frozen) | 6 | *Anaplasma, Ehrlichia* | | Molecular analysis | Parasitology lab of the IPVDF |
|  | | Black-browed albatross  *Thalassarche melanophris*  *(n = 29)* | | | Rehabilitation centers, Brazil (UNIVALI, CECLIMAR), and BAAP | | Blood (frozen and in ethanol) | 13 | *Anaplasma, Ehrlichia* | | Molecular analysis | Parasitology lab of the IPVDF |
|  | |  | | | Rehabilitation centers, Brazil (UNIVALI, CECLIMAR), and BAAP | | Liver (frozen) | 13 | *Anaplasma, Ehrlichia* | | Molecular analysis | Parasitology lab of the IPVDF |
|  | |  | | | Rehabilitation centers, Brazil (UNIVALI, CECLIMAR), and BAAP | | Spleen (frozen) | 2 | *Anaplasma, Ehrlichia* | | Molecular analysis | Parasitology lab of the IPVDF |
|  | |  | | | PMP-BS⁵, Brazil | | Liver (in RNA later) | 12 | *Plasmodium, Haemoproteus,* | | Molecular analysis | LABCAI of the Federal University of Santa Catarina state and parasitology lab of the IPVDF |
| **Procellariidae**  **(n = 173)** | Great shearwater  *Ardenna gravis*  *(n = 6)* | | | | Rehabilitation centers, Brazil (UNIVALI, CECLIMAR), and BAAP | | Blood (frozen and in ethanol) | 3 | *Anaplasma, Ehrlichia* | | Molecular analysis | Parasitology lab of the IPVDF |
|  |  | | | | Rehabilitation centers, Brazil (UNIVALI, CECLIMAR), and BAAP | | Liver (frozen) | 3 | *Anaplasma, Ehrlichia* | | Molecular analysis | Parasitology lab of the IPVDF |
|  | |  | | | Rehabilitation centers, Brazil (UNIVALI, CECLIMAR), and BAAP | | Spleen (frozen) | 3 | *Anaplasma, Ehrlichia* | | Molecular analysis | Parasitology lab of the IPVDF |
|  | | Sooty shearwater  *Ardenna grisea*  *(n = 2)* | | | Rehabilitation centers, Brazil (UNIVALI, CECLIMAR), and BAAP | | Liver (frozen) | 2 | *Anaplasma, Ehrlichia* | | Molecular analysis | Parasitology lab of the IPVDF |
|  | |  | | | Rehabilitation centers, Brazil (UNIVALI, CECLIMAR), and BAAP | | Spleen (frozen) | 2 | *Anaplasma, Ehrlichia* | | Molecular analysis | Parasitology lab of the IPVDF |
|  | | Cory's shearwater  *Calonectris borealis*  *(n = 10)* | | | Rehabilitation centers, Brazil (UNIVALI, CECLIMAR), and BAAP | | Blood (frozen and in ethanol) | 3 | *Anaplasma, Ehrlichia* | | Molecular analysis | Parasitology lab of the IPVDF |
|  | |  | | | Rehabilitation centers, Brazil (UNIVALI, CECLIMAR), and BAAP | | Liver (frozen) | 7 | *Anaplasma, Ehrlichia* | | Molecular analysis | Parasitology lab of the IPVDF |
|  | |  | | | Rehabilitation centers, Brazil (UNIVALI, CECLIMAR), and BAAP | | Spleen (frozen) | 4 | *Anaplasma, Ehrlichia* | | Molecular analysis | Parasitology lab of the IPVDF |
|  | | Cape petrel  *Daption capense*  *(n = 3)* | | | Rehabilitation centers, Brazil (UNIVALI, CECLIMAR), and BAAP | | Blood (frozen and in ethanol) | 2 | *Anaplasma, Ehrlichia* | | Molecular analysis | Parasitology lab of the IPVDF |
|  | |  | | | Rehabilitation centers, Brazil (UNIVALI, CECLIMAR), and BAAP | | Liver (frozen) | 1 | *Anaplasma, Ehrlichia* | | Molecular analysis | Parasitology lab of the IPVDF |
|  | |  | | | Rehabilitation centers, Brazil (UNIVALI, CECLIMAR), and BAAP | | Spleen (frozen) | 1 | *Anaplasma, Ehrlichia* | | Molecular analysis | Parasitology lab of the IPVDF |
|  | | Southern fulmar  *Fulmarus glacialoides*  *(n = 1)* | | | Rehabilitation centers, Brazil (UNIVALI, CECLIMAR), and BAAP | | Blood (frozen and in ethanol) | 1 | *Anaplasma, Ehrlichia* | | Molecular analysis | Parasitology lab of the IPVDF |
|  | | Southern giant petrel  *Macronectes giganteus*  *(n = 8)* | | | Rehabilitation centers, Brazil (UNIVALI, CECLIMAR), and BAAP | | Blood (frozen and in ethanol) | 6 | *Anaplasma, Ehrlichia* | | Molecular analysis | Parasitology lab of the IPVDF |
|  | |  | | | Rehabilitation centers, Brazil (UNIVALI, CECLIMAR), and BAAP | | Liver (frozen) | 5 | *Anaplasma, Ehrlichia* | | Molecular analysis | Parasitology lab of the IPVDF |
|  | |  | | | Rehabilitation centers, Brazil (UNIVALI, CECLIMAR), and BAAP | | Spleen (frozen) | 4 | *Anaplasma, Ehrlichia* | | Molecular analysis | Parasitology lab of the IPVDF |
|  | | Northern giant petrel   \| *Macronectes halli*  (n = 33) \| \| --- \| | | | Rehabilitation centers, Brazil (UNIVALI, CECLIMAR), and BAAP | | Blood (frozen and in ethanol) | 1 | *Anaplasma, Ehrlichia* | | Molecular analysis | Parasitology lab of the IPVDF |
|  | |  | | | Bird Island, South Georgia  2014/2015 season | | Blood (in FTA cards) and blood smears (on microscope slides) | 32 | *Plasmodium, Haemoproteus, Leucocytozoon, Borrelia burgdorferi s.l.* | | Molecular analysis and Blood smear | Molecular biology lab of the University of Exeter |
|  | |  | | | Rehabilitation centers, Brazil (UNIVALI, CECLIMAR), and BAAP | | Liver (frozen) | 1 | *Anaplasma, Ehrlichia* | | Molecular analysis | Parasitology lab of the IPVDF |
|  | |  | | | Rehabilitation centers, Brazil (UNIVALI, CECLIMAR), and BAAP | | Spleen (frozen) | 1 | *Anaplasma, Ehrlichia* | | Molecular analysis | Parasitology lab of the IPVDF |
|  | | White-chinned petrel  *Procellaria aequinoctialis*  (n = 61) | | | Rehabilitation centers, Brazil (UNIVALI, CECLIMAR), and BAAP | | Blood (frozen and in ethanol) | 17 | *Anaplasma, Ehrlichia* | | Molecular analysis | Parasitology lab of the IPVDF |
|  | |  | | | Bird Island, South Georgia  2014/2015 season | | Blood (in FTA cards) and blood smears (on microscope slides) | 32 | *Plasmodium, Haemoproteus, Leucocytozoon, Borrelia burgdorferi s.l.* | | Molecular analysis and Blood smear | Molecular biology lab of the University of Exeter |
|  | |  | | | Rehabilitation centers, Brazil (UNIVALI, CECLIMAR), and BAAP | | Liver (frozen) | 23 | *Anaplasma, Ehrlichia* | | Molecular analysis | Parasitology lab of the IPVDF |
|  | |  | | | Rehabilitation centers, Brazil (UNIVALI, CECLIMAR), and BAAP | | Spleen (frozen) | 9 | *Anaplasma, Ehrlichia* | | Molecular analysis | Parasitology lab of the IPVDF |
|  | | Atlantic petrel  *Pterodroma incerta*  (n = 1) | | | Rehabilitation centers, Brazil (UNIVALI, CECLIMAR), and BAAP | | Blood (frozen and in ethanol) | 1 | *Anaplasma, Ehrlichia* | | Molecular analysis | Parasitology lab of the IPVDF |
|  | | Soft-plumaged petrel  *Pterodroma mollis*  (n = 1) | | | Rehabilitation centers, Brazil (UNIVALI, CECLIMAR), and BAAP | | Blood (frozen and in ethanol) | 1 | *Anaplasma, Ehrlichia* | | Molecular analysis | Parasitology lab of the IPVDF |
|  | | Manx shearwater  *Puffinus puffinus*  (n = 42) | | | Rehabilitation centers, Brazil (UNIVALI, CECLIMAR), and BAAP | | Blood (frozen and in ethanol) | 17 | *Anaplasma, Ehrlichia* | | Molecular analysis | Parasitology lab of the IPVDF |
|  | |  | | | Rehabilitation centers, Brazil (UNIVALI, CECLIMAR), and BAAP | | Liver (frozen) | 27 | *Anaplasma, Ehrlichia* | | Molecular analysis | Parasitology lab of the IPVDF |
|  | |  | | | Rehabilitation centers, Brazil (UNIVALI, CECLIMAR), and BAAP | | Spleen (frozen) | 10 | *Anaplasma, Ehrlichia* | | Molecular analysis | Parasitology lab of the IPVDF |
| **Oceanitidae**  **(n = 5)** | | Wilson's storm petrel  *Oceanites oceanicus*  (n = 5) | | | Rehabilitation centers, Brazil (UNIVALI, CECLIMAR), and BAAP | | Blood (frozen and in ethanol) | 5 | *Anaplasma, Ehrlichia* | | Molecular analysis | Parasitology lab of the IPVDF |

Legend: Total number of birds do not correspond to the sum of samples listed because different sets of tissues were available for analysis for each individual. Acronyms used: BAAP = Banco Nacional de Amostras Biologicas de Albatrozes e Petreis, CECLIMAR = Centro de Estudos Costeiros, Limnológicos e Marinhos, IPVDF = Instituto de Pesquisas Veterinárias Desidério Finamor, LABCAI = Laboratório de Biomarcadores de Contaminação Aquática e Imunoquímica, UNIVALI = Universidade do Vale de Itajaí.
